# Did You Ever Hear the One About the Horse that Could Count?

**DOI:** 10.3389/fpsyg.2012.00357

**Published:** 2012-09-21

**Authors:** Michael J. Beran

**Affiliations:** ^1^Language Research Center, Georgia State UniversityAtlanta, GA, USA

“Those who don’t know history are destined to repeat it.”

*(Edmund Burke)*

It has become increasingly, and sometimes frustratingly, clear that in the past few years some researchers working with non-human animals either have forgotten (or were never taught) the perils of inadvertent cuing. I wrote this article after completing another journal review in which the methodology involved an experimenter presenting two or more choices to an animal. The experimenter prepared the trial, presented it, watched the animal as it made its response, and then recorded that response. In this case, as in some others in our field, the test itself was creative, unique, and exciting, and the performance by the animals tested was adequate to suggest they might be doing something interesting and perhaps reflective of cognitive processing. But, the possibility that cuing might have occurred dampened my enthusiasm for the project, and dampened my spirits about the state and future of the field in general because too many papers get through the peer review process without having proper controls for cuing. In the interests of full disclosure, I cannot say I have always been perfect in preventing any chance of cuing in the tests I have done with animals, but I do worry that more and more often there is not even the recognition of the need to control for possible cuing in experiments assessing animal cognition.

When I started graduate school, the first project I worked on involved computerized testing of chimpanzees that were learning to match Arabic numerals to dots on the screen. They saw a numeral, and had to collect dots, one-at-a-time, until they indicated they thought they had the right number. If they were right, the computer played a tone, and if wrong, it played a buzz. My job was to give them a treat when they were right, and of course to also try to keep them engaged in the task in general. The first thing I was told, though, was “never look at the computer screen while they are working. If you do not know how they are doing until the computer tells you, you cannot cue them while they are working.”

Hence the problem when the experimenter watches the response. He or she knows the correct response, and almost certainly hopes the animal will make that response (otherwise, no publication, no degree, no grant, no tenure, etc.). In this case, *experimenter expectation* rears its ugly head, and cannot be controlled. It is not my intention in this article to “call out” any particular researcher or team of researchers, but it is critical to get the attention of those who are failing to design adequate methods for controlling inadvertent cuing. Some of these groups make (or, at least, report) almost no attempt to control for cuing, whereas others do implement some design aspects to address cuing, but not enough. Sometimes, one reads in manuscripts or in published articles something like “the experimenter looked straight ahead, did not look at the animal, did not respond to the animal, or otherwise did not give any feedback to the animal during its response.” Plain and simple, this is not possible to conclude. This was exactly the lesson of the horse named Clever Hans, a lesson that is now more than 100 years old, but also one that seems to be increasingly forgotten.

Clever Hans was, indeed, an incredibly clever animal. The problem was that he was clever in ways not related to the apparent intellect that first drew attention to him. Initially, it was believed that Hans was capable of all kinds of mathematical and computational feats (pun intended, given that Hans responded to questions by tapping his hoof). He consistently provided the correct answer to all manner of questions. And, initially, some of the foremost experts on animal behavior validated his performance as reflecting true cognitive skill. However, this was not true. Instead, the cleverness of Hans was reflected in his acute sensitivity to subtle cues given by those who asked him questions. The mystery was solved when it became clear that Hans only answered questions correctly when the people asking the question and watching his answer also knew the answer themselves (Pfungst, 1911). Hans was using tension, concentration, relaxation, some changes in posture, and other similar kinds of bodily cues that people exhibited as he was responding. Thus became the critical lesson of Clever Hans – if you know the answer, you should not ask the question and score the response given by the participant. If you do, the possibility for cuing exists, and the potential for erroneous interpretations of the responses of subjects also exists.

This concern about cuing, in fact, partially inspired the development of the one of the most important, and longest lasting, apparatus used in comparative psychology – Harlow’s Wisconsin General Test Apparatus (WGTA; Harlow and Bromer, 1938; Harlow, 1949). One point of the WGTA (and of even earlier apparatus that were precursors to the WGTA) was to make sure that the animal could not see the experimenter at all during the set-up of trials and during its own response. Instead, the experimenter viewed the animal in a one-directional manner, preventing any possible cues from occurring. Subsequent use of versions of the WGTA occurred in many animal laboratories, and the development of computerized testing with non-human primates (e.g., Rumbaugh et al., 1989) and then other species also was at least partly due to the desire to eliminate the potential for cuing of subjects. And, of course, test boxes of other kinds (e.g., Skinner boxes) used with pigeons, rats, and other animals eliminated this concern as well.

Note that this is not a concern only for animal researchers. It is a possibility with any test subject. All too often in developmental studies, for example, researchers act as if such cues are not possible with human children (or, for that matter, with adult human participants). And, in comparative psychology, especially in tests of comparative cognition, the methods are often adopted and adapted from developmental psychology. Hence, the problem compounds. Even worse is when research teams, when asked why they do not have adequate controls, respond by saying “this is how it is done with children, or by group X who did it before us with species Y.” This is an entirely inadequate and misguided justification. My contention is that any study that fails to control for cuing is flawed, and it should not be replicated, at least with regard to the methodological details that allowed for the potential cuing to occur.

One might ask whether the problem is really that worrisome, and the answer is a resounding yes. First, many empirical comparative studies looking at cognitive processes involve only a small number of subjects, and these studies are rarely replicated by other laboratories or with other subjects (see Agrillo and Miletto Petrazzini, 2012). So, the first report is often the only one, and a positive report of some new behavioral phenomenon is likely to be highly cited, and highly influential on theory and subsequent work in that topic area. But if the possibility of cuing exists, we are then stuck with equivocal data, and perhaps erroneous conclusions.

The problem of cuing can be even worse when the phenomenon of interest might have practical, real-world implications. One of the best examples of this comes from a recent paper by Lit et al. (2011). They tested whether the beliefs of human handlers could impact the behavior of scent dogs – dogs trained to provide critical services by finding drugs or explosives. When human handlers thought (incorrectly) that a site was baited with a relevant scent, they reported that the dogs more often alerted at those locations. In other words, Lit and colleagues showed that the handlers’ beliefs affected what the dogs did.

The solution is simple: remember Clever Hans! Teach students his story, and engrain in them the need to, at minimum, run control trials/sessions in which possible cuing is prevented, so that they can see whether responding remains the same as when such controls are not present. Even better, eliminate possible cuing totally, through the use of multiple experimenters who either see what the animal does (but do not know what it *should* do) or who prepare trials but then do not see what response the animal makes. This will let us increase our confidence that the animal sitting across from us is responding on the basis of its own learning, or its own “thinking,” rather than on the basis of adjusting its responses based on how we are reacting to what it is doing. By doing this, we will put the Clever Hans Effect back in the barn, and out of view, while keeping Clever Hans the reminder in full view.
